# No evidence for motor-recovery-related cortical connectivity changes after stroke using resting-state fMRI

**DOI:** 10.1152/jn.00148.2021

**Published:** 2021-12-29

**Authors:** Meret Branscheidt, Naveed Ejaz, Jing Xu, Mario Widmer, Michelle D. Harran, Juan Camilo Cortés, Tomoko Kitago, Pablo Celnik, Carlos Hernandez-Castillo, Jörn Diedrichsen, Andreas Luft, John W. Krakauer

**Affiliations:** ^1^Brain Physiology & Stimulation Laboratory, Department of Physical Medicine & Rehabilitation, Johns Hopkins University, Baltimore, Maryland; ^2^Department of Neurology, University Hospital Zurich, Zurich, Switzerland; ^3^Brain and Mind Institute, Western University, London, Ontario, Canada; ^4^Department of Neuroscience, Johns Hopkins University, Baltimore, Maryland; ^5^Malone Center for Engineering in Healthcare, Johns Hopkins University, Baltimore, Maryland; ^6^Cereneo Vitznau-Center for Neurology and Rehabilitation, Vitznau, Switzerland; ^7^Department of Neurology, Johns Hopkins University, Baltimore, Maryland; ^8^Burke Neurological Institute and Weill Cornell Medicine, White Plains, New York

**Keywords:** cortical reorganization, functional connectivity, motor recovery, resting-state imaging, stroke

## Abstract

It has been proposed that a form of cortical reorganization (changes in functional connectivity between brain areas) can be assessed with resting-state (rs) functional MRI (fMRI). Here, we report a longitudinal data set collected from 19 patients with subcortical stroke and 11 controls. Patients were imaged up to five times over 1 year. We found no evidence, using rs-fMRI, for longitudinal poststroke cortical connectivity changes despite substantial behavioral recovery. These results could be construed as questioning the value of resting-state imaging. Here, we argue instead that they are consistent with other emerging reasons to challenge the idea of motor-recovery-related cortical reorganization poststroke when conceived of as changes in connectivity between cortical areas.

**NEW & NOTEWORTHY** We investigated longitudinal changes in functional connectivity after stroke. Despite substantial motor recovery, we found no differences in functional connectivity patterns between patients and controls, nor any changes over time. Assuming that rs-fMRI is an adequate method to capture connectivity changes between cortical regions after brain injury, these results provide reason to doubt that changes in cortico-cortical connectivity are the relevant mechanism for promoting motor recovery.

## Graphical abstract

**Figure:**
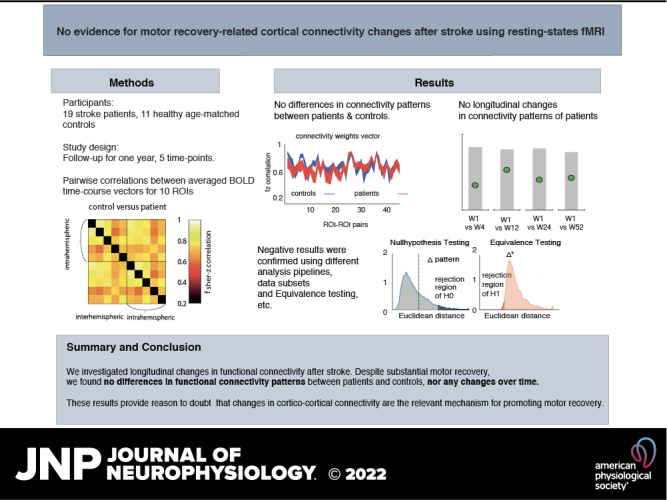

## INTRODUCTION

Spontaneous recovery occurs in almost all patients with stroke within the first months of the insult. Although the physiological changes associated with spontaneous recovery in humans remain largely unknown, data from animal models have led to the notion of cortical reorganization as a potential key mechanism (1–3).

In the literature, the term “cortical reorganization” is loosely defined, referring to any number of structural/physiological changes after injury. These changes can span the micro-, meso-, and macroscale, including synaptogenesis, axonal sprouting, expansion of cortical activation maps, and changes in cortico-cortical connectivity. We have argued elsewhere that the term “functional reorganization” should be reserved for those changes, including new or altered cortico-cortical connections, which are causally related to or at least correlated with recovery (4).

Evidence for functional reorganization in the form of new cortico-cortical connections after stroke comes primarily from animal studies of axonal sprouting. For example, Overman et al. (5), in a mouse cortical stroke model, generated sprouting of axonal connections within ipsilesional motor, premotor, and prefrontal areas by blocking of a growth inhibitor (epinephrine A5). Similar results were reported for the neuronal growth factor GDF10 (6). Critically, however, in these studies, no direct test of the causal relevance of axonal sprouting for motor improvement was performed. Despite the weak evidence for behaviorally relevant cortical connectivity changes in animal models after stroke, there has been a widespread interest in identifying similar changes in the human brain, with noninvasive techniques. One prominent method is to measure interregional connectivity with resting-state functional MRI (rs-fMRI; 7, 8). It relies on correlations between time series of fMRI activity recorded while the subject is lying in the scanner without performing a task. These correlations are commonly regarded as a measure of “functional connectivity” (9, 10). In the context of stroke recovery, it has been suggested that functional reorganization can be detected as a change in such correlations/functional connectivity patterns (11). Specifically, for poststroke recovery of hemiparesis, the advantage of task-free resting-state over task-based fMRI is that it avoids the performance confound (12, 13); the connectivity measures are not biased by the inability of patients to match control performance.

To date, results from rs-fMRI studies of functional connectivity changes underlying motor recovery have been mixed. Although rs-fMRI studies have frequently found changes in interhemispheric connectivity patterns after stroke (14–16), the direction of these changes and their correlations with behavior have been inconsistent. There have also been recent results that failed to find cortical connectivity changes in poststroke motor recovery (17).

There are many potential reasons for these inconsistencies in rs-fMRI findings. If patients with cortical lesions are included in the study design, it is possible to misinterpret changes in connectivity as functional reorganization when they may just be a reactive response to the lesion. In addition, studies have used different analysis protocols and measures to quantify changes in connectivity, making integration of evidence difficult. Third, the majority of currently available studies have been cross-sectional, but it is essential to evaluate changes in connectivity longitudinally if questions concern recovery.

To address these issues, we report the results of a longitudinal rs-FMRI study of stroke recovery in patients with hemiparesis after subcortical stroke. Only patients with subcortical lesions were included so that any changes in cortical connectivity could not be attributed to the presence of the lesion itself. Because of considerable variation in the analysis approaches reported in the literature, in addition to our primary analysis, we also applied two additional preprocessing procedures, report results from an individual M1-M1 ROI analysis, and replicated the analysis approach used in the largest longitudinal resting-state stroke study of motor recovery published to date (16).

## METHODS

The resting-state data set presented here were acquired from a natural history study investigating upper extremity recovery after stroke (Study of Motor Acute Recovery Time course after Stroke; SMARTS). As part of the study, a range of behavioral, physiological, and imaging measurements was obtained. Details of the behavioral characterization of the patients have been published elsewhere (18–20).

### Patients

**Figure 1.**
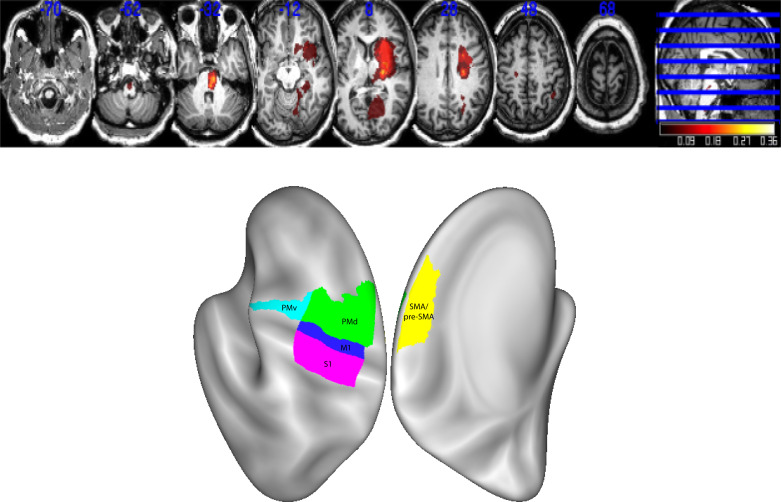
Lesion distribution (*n* = 19). Averaged lesion distribution mapped to MNI space with lesions flipped to one hemisphere. *Bottom*: the surface-based rendering of the regions of interest (ROI). Note that there was one patient with a small bilateral stroke but had only unilateral symptoms. PMd, dorsal premotor cortex; PMv, ventral premotor cortex; SMA, supplementary motor area.

As we were interested in cortical connectivity changes after stroke, we wanted to avoid confounding results due to cortical damage, and so limited our analysis to a subset of 19 patients with a subcortical lesion to the corticospinal tract/no cortical lesion within the motor system (6 females; mean age 59 ± 12 yr, 15 right handed). Major inclusion criteria were: first-ever clinical apparent ischemic stroke, proven by a positive diffusion weighted imaging (DWI) lesion within the previous 2 wk; unilateral upper extremity weakness (Medical Research Council muscle weakness scale <5); and the ability to give informed consent. Patients were excluded for one or more of the following reasons: initial impairment too mild (Fugl–Meyer score upper extremity >63/66), age ≤21 yr, and hemorrhagic stroke (20). The selected patients had lesions in the corticospinal tract above the crossing in the pyramid. Demographics are described in Table 1; more detailed information about lesion distribution is shown in Fig. 1.

**Table 1.** Patient demographics and overall session count

Patient No.	Age	Gender	Handedness	LesionSide	First FM-UE	First ARAT	Session
1	57	M	Right	Left	58	56	5
2	53	F	Right	Right	64	57	4
3	65	M	Right	Right	30	21	4
4	66	M	Right	Right	66	56	3
5	66	F	Right	Right	60	55	5
6	71	M	Right	Right	4	0	3
7	46	M	Left	Left	4	0	4
8	46	M	Right	Right	49	52	5
9	67	F	Right	Left	16	2	4
10	56	M	Right	Right	64	57	4
11	59	F	Right	Left	60	57	5
12	64	M	Left	Right	63	57	4
13	74	M	Left	Left	5	0	5
14	80	F	Right	Left	9	56	5
15	64	F	Right	Right	58	39	5
16	22	M	Right	Left	63	56	5
17	53	M	Left	Left	30	39	5
18	54	M	Right	Right	59	57	5
19	58	M	Right	Right	61	56	4

Please note that subjects have a FM-UE score >63. This is explained by clinical recovery between enrolment and the first measurement time point. First FM-UE, first recorded Fugl–Meyer score upper extremity; first ARAT, first recorded Arm Research Action Test; session, number of time points.

In addition, 11 healthy age-matched control participants (4 females; mean age 65 ± 8 yr; all right handed) were tested at the same time-points. It should be noted that controls were on average older than the patients.

The study was carried out in accordance with the Declaration of Helsinki and approved by the respective local ethics committee of the participating recruiting centers of SMARTS (Johns Hopkins University, Baltimore, MD; Columbia University, New York, NY; University Hospital Zurich, Switzerland). All participants gave written informed consent.

### Study Design

Patients were enrolled in the study within the first 2 wk after stroke and followed up over a 1-year period at five time-points: early subacute stage *W1*: *weeks 1–2* (10 ± 4 days), *W4*: *weeks 4–6* (37 ± 8 days), *W12*: *weeks 12–14* (95 ± 10 days), *W24*: *weeks 24–26* (187 ± 12 days), and *W52*: *weeks 52–54* (370 ± 9 days). During each visit, the following clinical parameters were assessed: Fugl–Meyer score upper extremity (FM-UE, maximum score 66; 21), Action Research Arm Test (ARAT, maximum score 57; 22). Hand strength and individuation ability were measured using a custom-made hand device (20). The FM-UE and ARAT are widely used to assess motor deficits after stroke and can capture different aspects of recovery: higher FM-UE scores represent normal reflex activity, fewer muscular coactivations, and coordination and higher joint mobility thought to be equal to “true” resolution of impairment; higher ARAT scores are achievable with compensatory strategies, thus correlating closer with activities of daily living. Measuring hand strength offers a third dimension of recovery that is only partially captured within the FM-UE and ARAT.

### Image Acquisition

Participants were scanned with a 3 T Achieva Philips system. Scans were obtained with a 32-channel head coil, using a two-dimensional echo-planar imaging sequence (TR = 2.00 s, 35 slices, 210 volumes/run, slice thickness = 3 mm, 1 mm gap, in-plane resolution = 3 × 3 mm^2^). Each resting-state scan was 7.12 min long. Participants were instructed to lie still and visually fixate on a central white cross displayed on a computer monitor.

Structural images for atlas transformation and lesion definition were acquired with a T1-weighted anatomical scan (3D MP-RAGE sequence, TR/TE = 8/3.8 ms, FOV = 212 × 212 mm, matrix 96 × 96, 60 slices, slice thickness = 2.2 mm). Finally, for each participant, a diffusion-weighted imaging (DWI/ADC) image (TR = 2.89 s, 30 slices, slice thickness = 5 mm, FOV = 240 × 240 mm) was acquired to define lesion boundaries.

### Imaging Analysis

#### Preprocessing of rs-fMRI time series.

Rs-fMRI has a relatively low signal-to-noise ratio. Nonneuronal processes, such as sensor noise, head motion, cardiac phase, and breathing, account for a considerable part of the variance of the raw signal (23). It has been argued that markers for the reliability of the sampled rs-fMRI data are missing and that justification for choice of preprocessing steps is often not given (24, 25). We, therefore, conducted three different procedures for noise reduction and then compared split-half reliability for the whole connectivity pattern in controls to determine which steps provided higher reliability (see under *Preprocessing approaches*). Comparing three different preprocessing pipelines added an extra layer of reliability to our results that most clinical resting-state studies do not provide.

#### Lesion definition.

Lesion boundaries were defined as an intensity increase of ≥30% on DWI images, and, in a second step, manually modified by a neuroradiologist and a neurologist using RoiEditor, see Fig. 1 for averaged lesion distribution map and surface-based rendering of the ROI.

#### ROI definition.

We chose five motor areas (S1 = primary somatosensory cortex, M1 = primary motor cortex, PMd = dorsal premotor cortex, PMv = ventral premotor cortex, SMA = supplementary motor area) as regions of interest (ROI). We restricted our analysis to only include ROIs that are known to be relevant for hand motor function, have been hypothesized to play a role in motor recovery, and are directly or indirectly connected to corticospinal projections. We based our choice on extensive anatomical studies regarding motor recovery after stroke in rodents and nonhuman primates (4, 26, 27). Individual T1-images were used to delineate pial gray matter and gray matter-white matter boundaries using FreeSurfer software (28). The cortical surfaces were aligned across participants based on the sulcal-depth and local curvature maps. Probabilistic cytoarchitectonic maps (29) aligned to the group average surface were then used to define ROIs, first on the individual surface and then back projected into the subject-native space.

The ROIs were defined as follows: M1, surface nodes with the highest probability for Brodmann area (BA) 4. To increase specificity for processes related to recovery of hand function, this ROI was limited to 2 cm above and below the hand knob (30). S1, nodes in the hand region in S1 were isolated using BA 3a and 3b, 1 and 2.2 cm above and below the hand knob. PMd, nodes with highest probability in BA6, above middle frontal sulcus, but on the lateral surface of the hemisphere. PMv, nodes with the highest probability in BA6, below the middle frontal sulcus. SMA, nodes with the highest probability in BA6 on the medial surface of the brain. This ROI therefore includes SMA and preSMA (31).

### Functional Connectivity Analysis

For each ROI, the time series for all voxels within the ROI were extracted and averaged, resulting in a single BOLD time-course vector for each of the 10 ROIs across the two hemispheres (left-S1, left-M1, left-PMd, left-PMv, left-SMA, right-S1, right-M1, right-PMd, right-PMv, right-SMA). Pairwise correlations between averaged BOLD time-course vectors for the different ROIs were computed, and Fisher-Z was transformed to conform better to a normal distribution, resulting in a 10 × 10 matrix of connectivity weights (Fig. 3). The matrix thus represents the connectivity weights between all possible ROIs for a patient: 10 intrahemispheric ROI pairs, each within the ipsilesional and contralesional hemispheres, respectively, and 25 interhemispheric ROI pairs between the ipsilesional and contralesional hemispheres (overall 45 connectivity weights for all ROI pairs). For the rest of this manuscript, this vectorized, Fisher-Z-transformed correlation matrix will be referred to as the full connectivity pattern, whereas the corresponding intra- and interhemispheric subsets of the matrix will be referred to as the intrahemispheric contralesional (1 × 10 vector), intrahemispheric ipsilesional (1 × 10 vector), and interhemispheric connectivity patterns (1 × 25 vector), respectively. These connectivity patterns were estimated independently for each session and patient. Connectivity patterns for controls were estimated similarly, with the exception that intrahemispheric connectivity patterns were averaged across both hemispheres.

#### Intrasession reliability.

To estimate the reliability of our measurements within sessions, connectivity patterns were computed as described above for the first 100 volumes and the second 100 volumes independently and correlated with each other to calculate split-half reliabilities.

#### Preprocessing approaches.

The intrasession reliability measurement also allowed us to compare three different preprocessing procedures:

*First preprocessing procedure* (P1): We removed the first 10 volumes of the functional data, then performed correction for the timing of slice acquisition, motion correction, brain extraction, linear trend removal, and temporal filtering (band pass, 0.01–0.08 Hz), using FSL [FMRIB Software Library (FSL), Oxford University, Oxford, UK]. Our analysis was carried out in the native space, and no spatial smoothing was applied. Linear regression was used to remove signal correlated with the global mean signal and the average time series in the cerebral white matter and cerebrospinal fluid (32).

*Second preprocessing procedure* (P2): Here, we used an independent component analysis (ICA) approach using FSL MELODIC for artifact reduction (33). Again, we removed the first 10 volumes of the functional data. We applied motion correction and brain extraction. Probabilistic independent component analysis was conducted to denoise the individual data by removing components such as head motion, scanner artifacts, and physiological noise. Noise components were classified using FMRIB’s ICA-based Xnoiseifier (34), which attempts to autoclassify ICA components into “good” versus “bad” components. The “bad” components were then removed from the functional data.

*Third preprocessing procedure* (P3): Recent benchmarks showed that although no method completely abolishes noise effects, the use of ICA-AROMA including global signal regression (GSR) showed the best performance in several tests (35), and it also led to more reliable group differences (36). In brief, the P3 core processing pipeline included the following steps: *1*) removal of the first four volumes of each acquisition; *2*) realignment of all volumes to a selected reference volume using mcflirt (37); *3*) demeaning and removal of any linear or quadratic trends; *4*) coregistration of functional data to the high-resolution structural image using boundary-based registration (38); *5*) temporal filtering using a first-order Butterworth filter with a passband between 0.01 and 0.08 Hz. We did not apply slice timing correction during preprocessing, as recent data suggest that the interpolation that occurs may artificially reduce motion estimates (39); *6*) spatial smoothing using a 6-mm FWHM Gaussian kernel (required to be used in Aroma). *7*) ICA-AROMA denoising that includes the automatic classification of noise components in the individual time series and the regression of the selected components. In the regression the mean signal from WM, CSF, and Global Signal were included.

To determine which procedure would provide a more stable result, we calculated the split-half reliability of the ROI-ROI connectivity weights for the whole connectivity pattern over time in controls only.

All procedures led to good intrasession reliability on average (P1 = 0.64, CI 0.60–0.66; P2 = 0.62, CI 0.57–0.66; P3 = 0.60, CI 0.56–0.64) but showed no significant difference [χ^2^(2) = 3.242, *P* = 0.198], whereas no consistent change over time was found for either procedure by itself [P1: χ^2^(4) = 2.834, *P* = 0.684; P2: χ^2^(4) = 3.007, *P* = 0.557; P3: χ^2^(4) = 1.221, *P* = 0.875]. Because of the nominal higher intrasession reliability, we conducted all analyses after noise correction using the P1 procedure.

### Difference between Connectivity Patterns in Patients and Controls

In the early subacute recovery period (*W1*), stroke-related damage could alter connectivity patterns in patients in two distinct ways: *1*) The connectivity pattern could remain the same but overall connection strengths might be increased or decreased, resulting in connectivity patterns in patients DC-shifted but otherwise identical to control patterns. This would indicate that a canonical pattern of connectivity between motor ROIs in healthy people is simply upregulated- or downregulated after stroke, either due to maladaptation or compensation for damage. *2*) Stroke-related damage might alter connectivity weights among only a few select ROIs, e.g., either between ROIs within one hemisphere or across hemispheres. This would alter the shape of the connectivity patterns in patients in comparison with controls. As we wanted to be sensitive to both kinds of connectivity pattern change, the appropriate statistical test would be a MANOVA between patient and control connectivity patterns. However, due to insufficient degrees of freedom in performing such an analysis (the number of connectivity weights exceeds the number of patients and controls), we instead opted for a permutation test with Euclidean distance as a measure of dissimilarity between patient and control connectivity patterns, as it is sensitive to both shape and scaling changes in connectivity patterns.

#### Permutation tests.

To test against the null hypothesis of no difference between controls and patients, we performed a permutation test. We first identified patients and controls who had estimates of connectivity patterns for each week in question. We estimated Δpattern as the Euclidean distance between the average connectivity pattern for patients and the average connectivity pattern for controls. We then shuffled group assignment labels for connectivity patterns 10,000 times, randomly assigning connectivity patterns to “controls” or “patients.” From the shuffled data, we again calculated the Euclidean distance between the average connectivity pattern for patients and controls based on this new assignment. By repeatedly shuffling and computing Euclidean distances, we obtained an estimate of the empirical null distribution of Δpattern, e.g., the expected distribution if there was no real difference between the two groups. It is important to note that Euclidean distances (like *F*-statistics) are nonnegative, and that the estimate is systematically biased, e.g., it is larger on average than the “true” distance between controls and patients in the population. The measured Δpattern was then compared against this null distribution, and the relative proportion of simulations that showed a larger distance was used as a *P* value, the probability that the distance between the mean control and patient pattern would be equal or larger than the measured distance by pure chance. This analysis was carried out independently for the full, intrahemispheric ipsilesional, intrahemispheric contralesional, and interhemispheric connectivity patterns.

In case of nonsignificant results, we also conducted an Equivalence test (40). For each patient and control, we first calculated the deviation from their respective mean connectivity pattern. As for the null-hypothesis, we then again shuffled group assignment labels for connectivity patterns 10,000 times. For evaluating a specific alternative hypothesis, however, we added a random pattern to the patient group (injected a ground truth difference). The pattern was normally distributed across all connection weights and the pattern vector had a length of Δ*. For each simulation, we estimated Δpattern. This estimated distance was usually larger than Δ*, as the difference between the groups is also driven by random sampling error. We then reported the *d*, for which we only had a *P* = 0.05 chance of observing a value of the empirical Δpattern (or smaller). To express the effect size of this alternative hypothesis (which we could reject with *P* < 0.05), we calculated the average univariate Cohen’s *d* effect size for each connection. Note that a multivariate effect-size was not easily accessible, for the same reason that we could not employ a MANOVA.

Although, on average, connectivity patterns for patients did not differ from controls, individual patients could exhibit idiosyncratic connectivity patterns owing to the heterologous distribution of lesions locations in the cohort. Thus, early subacute (*W1*) stage changes in connectivity patterns might result in an increase in variability in within-group connectivity patterns. To determine whether this was the case at *W1*, we computed the average Euclidean distances between each patient’s connectivity pattern and the patients’ mean connectivity pattern (*W1* P_variability). Similarly, we computed the average Euclidean distance between each individual control pattern and the controls’ mean connectivity pattern (*W1* C_variability). The differences between these two served as a measure of increased or decreased variability in the patients (P_variability-C_variability = Δvariability). We then repeated the permutation test to generate a null distribution of the difference in variability to test the significance of Δvariability.

### Changes in Connectivity Patterns over Time during Recovery

**Figure 2.**
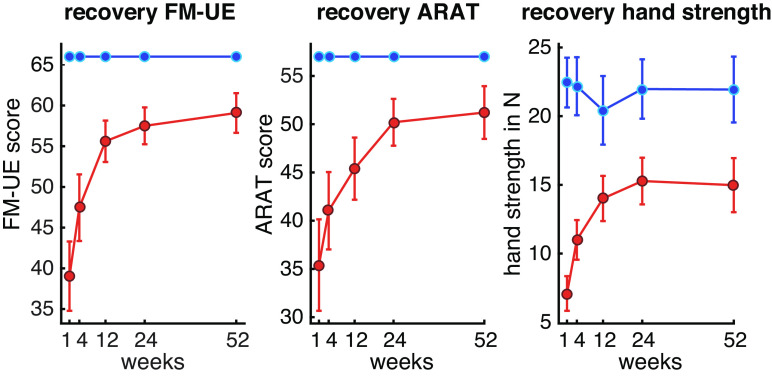
Recovery of upper extremity deficits after stroke over 1 year. For all behavioral assessments, the largest changes in recovery were seen within the first 3 mo. Patients reached a plateau at 6 mo and, on average, remained impaired compared with controls at all time-points. Red lines, patients; blue lines, controls; FM-UE, Fugl-Meyer score upper extremity; ARAT, Arm Research Action Test.

As patients in our cohort demonstrated substantial improvements of upper extremity deficits in the year after stroke (Fig. 2), we were interested to see whether there were concomitant longitudinal changes in connectivity patterns. To determine this, we performed two separate but related analyses. First, we independently compared differences in patient connectivity patterns from *W1* to all consecutive weeks using Euclidean distances (Δweek from *W1* to *W4*, *W12*, *W24*, and *W52*) to determine how far connectivity patterns diverged over the year from the pattern in the early subacute poststroke stage. The same was done for control connectivity patterns to establish intersession stability.

Second, we compared patient’s connectivity patterns for all five measurement sessions against the control connectivity patterns to determine how the patient patterns changed longitudinally in reference to controls (Δpattern for *W1*, *W4*, *W12*, *W24,* and *W52*). Both these analyses were performed using Euclidean distance and permutation testing in the same way as for estimating differences in connectivity patterns in the *W1* recovery stage.

To assess whether individual idiosyncratic patterns might show a change over time that could underlie recovery, we analyzed individual connectivity pattern changes for a subgroup of patients with all five time-points (10 patients) by comparing pattern variability in the early subacute stage against all other time-points (Δweek_variability for *W1*_*W4*, *W1*_*W12*, *W1*_*W24*, and *W1*_*W52*) and performing an ANOVA with the factor Weeks. In addition, we compared idiosyncratic variability between groups at all time-points.

### Alternative Metrics to Calculate Functional Connectivity

Because changes in functional connectivity between the two primary motor cortices have been the ones most consistently reported in the literature, we also explicitly looked at changes of M1-M1 connectivity weights.

We additionally analyzed our data set using a metric of functional connectivity that was proposed in the largest longitudinal resting-state stroke study to date with cortical and subcortical lesions, which reported changes of M1 interhemispheric connectivity. The metric has been called relative connectivity (RelCon) and is claimed to have low sensitivity to the temporal signal-to-noise ratio and signal amplitude fluctuations while maintaining a high sensitivity to meaningful signal changes, therefore, offering an advantage, e.g., in the analysis of data sets acquired with different scanners (41). RelCon looks at interhemispheric connectivity of M1 in relation to intrahemispheric connectivity of M1.

To calculate the interhemispheric RelCon for ipsilesional and contralesional sensorimotor cortex (SM1), the correlation between time series of all possible pairs of voxels is calculated (all voxels SM1_ipsilesional-contralesional_). The average of the interhemispheric connectivity for SM1_ipsilesional-contralesional_ is then calculated relative to the within connectivity of the ipsilesional SM1 (divided by the average correlation of all voxel within SM1_ipsilesional_).

This metric was tested on different real and simulated data sets and showed superior results compared with other absolute connectivity measures (absolute meaning connectivity measures that do not relate interhemispheric ROI-to-ROI connectivity weights to the average within correlation of the ipsilesional ROI itself).

Based on the reported method, we calculated the RelCon for interhemispheric SM1 connections in our data set.

### Statistical Analysis

Changes of behavioral measures in patients over time were analyzed using a mixed-effects ANOVA, with Week (*W1*–*W52*) as a fixed factor and Subject as a random factor. As ∼11% of the sessions were missing, we used the lme4 toolbox in R (42) to fit the unbalanced mixed-effects design. Rather than *F* values, statistical tests for main effects and interactions are reported using a χ^2^ approximation. Behavioral measures of patients and controls at *W1* were compared using a two-tailed *t* test.

Intrasession reliability was analyzed by computing split-half correlations (Pearson’s correlation) for each single week and individual patient/control, as well as looking at the averaged split-half correlation for all weeks together. Reliability between groups was compared using a mixed-effects ANOVA, with Group (patients vs. controls) and Week (*W1*–*W52*) as fixed and *Subject* as a random factor. This was done for all connections, as well as subsets only including interhemispheric, intrahemispheric ipsilesional, or contralesional ROIs.

Changes of interhemispheric M1-M1 connectivity weights over time between patients and controls were analyzed using a mixed-effects model, with either Group (patients versus controls) or Week (*W1*–*W52*) as fixed and Subject as a random factor. In addition, we tested changes over time in the subgroup of patients or controls with Week (*W1*–*W52*) as a fixed factor, separately. Alternative metrics reported in the study by Golestani et al. (16) were analyzed in the same way.

Results were considered significant at *P* < 0.05. Mean values are reported ± standard deviation unless stated otherwise.

### Data and Code Availability

The complete data set will be available upon request. All analysis was performed using built-in and custom-written MATLAB and R scripts that can be found at https://github.com/MeretBran/smarts_restingstate.

## RESULTS

The main goal of this study was to determine whether recovery from motor impairment following stroke was associated with systematic changes in cortical connectivity. Our two main questions were *1*) Is there a mean difference in the connectivity pattern between five motor regions (S1, M1, PMv, PMd, SMA) when comparing patients and age-matched controls at any time-point during stroke recovery? *2*) Is there a change in patients’ connectivity patterns over time that is related to motor impairment?

We analyzed data from 19 patients with subcortical stroke to the motor system and 11 healthy controls. Behavioral assessments and resting-state images were obtained at five different time-points over 1 year. Each patient completed on average 4.5 ± 0.7 sessions, with the overall experimental data being 89.5% complete (see also Table 1 for demographics and completed sessions in the methods ).

We begin by quantifying the extent of impairment and recovery of upper extremity deficits in our patients in the year following stroke.

### Patients Showed Substantial Clinical Recovery after Stroke

We measured initial impairment and subsequent recovery of the upper extremity using the upper extremity portion of the Fugl–Meyer score (FM-UE), the Action Research Arm Test (ARAT), and hand strength (20).

At *W1*, all behavioral measures indicated impairment of the upper extremity for patients relative to controls [FM-UE: *t*(13) = 5.487, *P* < 0.001, Cohen’s *d* = 0.97, ARAT: *t*(13) = 4.375, *P* < 0.001, Cohen’s *d* = 0.7, strength: *t*(13) = 5.195, *P* < 0.001, Cohen’s *d* = 2.3; Fig. 2]. These deficits recovered substantially over the course of 1 year, with the largest changes observed within the first 3 mo (week effect for FM-UE: χ^2^ = 24.865, *P* < 0.001; ARAT: χ^2^ = 13.942, *P* = 0.007; hand strength: χ^2^ = 13.419, *P* = 0.009). No significant changes were observed in controls for any of the three measures. It has to be noted that the patient cohort was heterogeneous in regard to their clinical deficits, with a subgroup of patients having only mild clinical impairments. To take this into consideration, we dichotomized our cohort using the average FM score in the first week as a cutoff. This resulted in a mild to moderately impaired group (*n* = 11; FM = 66-49) and a more severely impaired group (*n* = 8; FM = 30-0). The same analysis that was done for the whole cohort was done for both subgroups independently but did not yield different results.

### Connectivity Patterns across Sensorimotor Areas Were Reliable and Stable

**Figure 3.**
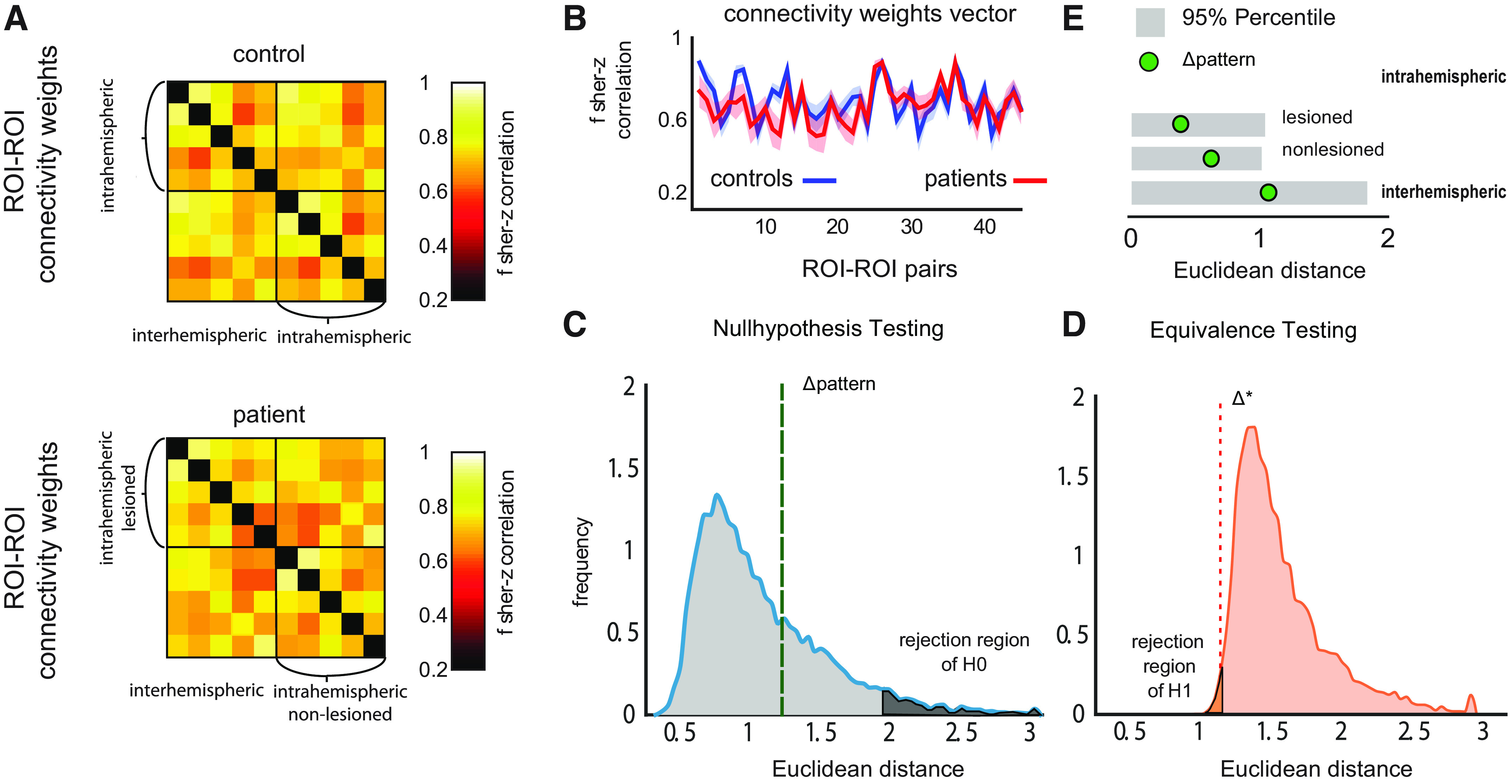
No systematic differences in connectivity patterns of patients and controls in the early subacute recovery period (*W1*). *A*: heat map representation of average connectivity weights for controls and patients at *W1*. The *y*- and *x*-axes show the five regions of interest (ROIs) (S1, M1, PMd, PMv, SMA) for the left and right hemisphere creating a connectivity matrix. Each square represents the connectivity weight for the respective ROI pairing. The diagonal (black) is missing, as it is the correlation of a ROI with itself. *B*: vectorized upper triangular part of the correlation matrix for the average full connectivity pattern of controls (blue line) and patients (red line). *C*: null hypothesis test. The distribution of the Euclidean distance between connectivity patterns for controls and patients, as expected under the null hypothesis of no differences (see methods). Green dashed line shows the observed Euclidean distance between patients and controls for *W1*. The value lies within the 95% of the null hypothesis and therefore outside of the rejection zone of H0. *D*: equivalence test. Distribution of Euclidean distance is under the assumption of a real difference of Δ* = 1.18. The real value (dashed line) lies in the rejection region of this test, such that we can reject any alternative hypothesis of Δ* > 1.18 at a significance level of *P* < 0.05. *E*: the measured Δpatterns (green circle) for the intrahemispheric ipsilesional, contralesional, or interhemispheric ROIs also always fell within the lower 95% percentile of the distribution under the null hypothesis (gray boxes), indicating no significant difference. PMd, dorsal premotor cortex; PMv, ventral premotor cortex; SMA, supplementary motor area.

We looked at changes in connectivity patterns (pattern of ROI-ROI connectivity weights) between five key sensorimotor areas to determine whether and how connectivity between these sensorimotor areas changed over the course of behavioral recovery. To determine the connectivity patterns, we calculated pairwise correlations between the averaged time series of BOLD activities between all possible ROI pairs to get a 10 × 10 matrix of connectivity weights (see methods). An average connectivity pattern for patients and controls is shown in Fig. 3*A*.

#### Intrasession reliability.

Connectivity patterns were highly reliable for both groups with moderate to good intrasession reliabilities (all connections, controls: *R* = 0.66, CI 0.62–0.71, patients: *R* = 0.70, CI 0.66–0.74). An unbalanced mixed-effects ANOVA (see methods) showed that the intrasession reliability was not significantly different between groups [χ^2^(1) = 1.0782, *P* = 0.2991] and showed no changes over time [controls: χ^2^(4) = 6.174, *P* = 0.187; patients: χ^2^(4) = 1.922, *P* = 0.75]. Patients always had slightly higher intrasession reliability than controls on average, although this difference was not significant and was possibly driven by outliers in the control group. Previous studies (e.g., Ref. 43) suggest that data quality could have been improved with a longer scan period; future studies should take this into account.

#### Intersession reliability.

Furthermore, connectivity patterns for controls were stable, showing no significant change over time (all connections: Δweek, *W1*_*W4* = 0.841, *P* = 0.662, Δ* > 0.68, *d* = 0.217; *W1*_*W12* = 0.689, *P* = 0.662, Δ* > 0.44, *d* = 0.165; *W1*_*W24* = 1.079, *P* = 0.489, Δ* > 0.97, *d* = 0.355; *W1*_*W52* = 1.059, *P* = 0.49, Δ* > 0.95, *d* = 0.392). Thus, for all subsequent analyses, connectivity patterns for controls were averaged over time-points.

We also confirmed that the connectivity pattern for controls reflected known anatomical connectivity (44). Within one hemisphere, the highest correlations were found between S1 and M1 (0.91 ± 0.47, Fisher-Z transformed), whereas the weakest correlation was found between M1 and PMv (0.58 ± 0.39). Between hemispheres, S1_right_-S1_left_ demonstrated the highest correlation (0.9 ± 0.43), whereas M1_right_-PmV_left_ showed a weaker correlation (0.59 ± 0.37). For correlations between hemispheres, homologous ROIs (e.g., M1-M1 or S1-S1) showed higher correlations of the BOLD time series compared with heterologous ROI-ROI connectivity weights (e.g., M1_right_-Pmv_left_ or S1_left_-Pmd_right_) as expected from interhemispheric neural recordings (45).

### There Were No Systematic Differences in Connectivity Patterns in the Early Subacute Recovery Period

If subcortical stroke leads to the disruption of cortical projections with subsequent early subacute reorganization of cortical circuits, one would expect that (on average) early subacute connectivity patterns of patients and controls would be different. Connectivity patterns for patients and controls were highly correlated in the early period after stroke (*W1*: *R* = 0.69).

To statistically test for significant differences between connectivity patterns, we used the Euclidean distance between the two groups’ mean patterns and compared it to a null distribution obtained by a permutation test (Fig. 3*A*, blue curve). We found no systematic difference between patients and controls at *W1* (Δpattern = 1.246, *P* = 0.296). This was also true when only considering intrahemispheric connections of either the ipsilesional (Δpattern = 0.552, *P* = 0.324) or contralesional side (Δpattern = 0.604, *P* = 0.340) or interhemispheric connections (Δpattern = 0.703, *P* = 0.674).

This null result of course does not mean that there was no difference between the connectivity patterns for patients and controls, only that the difference was not large enough for us to detect. To evaluate the statistical evidence against the alternative hypothesis that there is a true difference between the two groups, we conducted an equivalence test. We repeated the permutation test, this time inserting a true difference between the patterns of size Δ*. The estimated Δpattern for this alternative hypothesis tended to exceed the size of the true difference Δ*, simply due to the fact that the sampling error artificially causes some differences between two groups. From the simulations, however, it can be seen that based on our results, we can reject a difference of larger than Δ* > 1.18 at the significance level of *P* < 0.05. This pattern difference would translate to an average univariate effect size with Cohen’s *d* = 0.405 (a small effect size), distributed across the different connectivity weights. Although we, therefore, cannot exclude smaller and more focal average changes in the connectivity patterns, we have relatively clear evidence against a change in the connectivity pattern that could start to approach the effect size of the behavioral difference in strength (*d* = 2.3) or Fugl-Meyer scores (*d* = 0.97). We found similar results (interhemispheric: Δ* > 0.57, *d* = 0.242, ipsilesional: Δ* > 0.62, *d* = 0.464, contralesional: Δ* > 0.69, *d* = 0.471).

**Figure 4.**
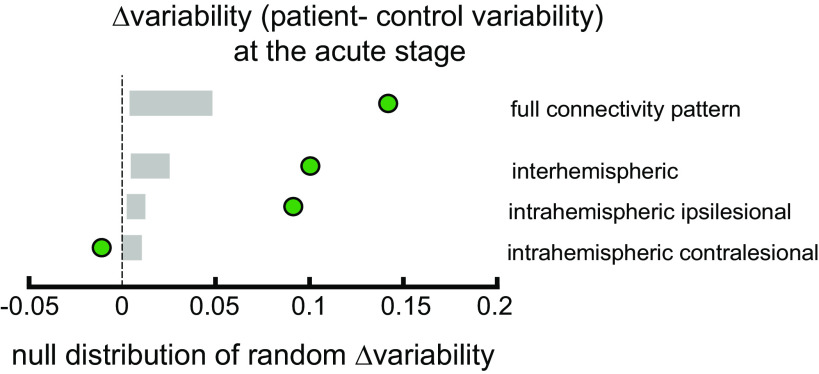
Patients showed a higher nonsystematic variability compared with controls at *W1* (Δvariability = green circle, 2.5%–97.5% range = gray boxes). Only for intrahemispheric contralesional regions of interest (ROIs) did patients show lower variability.

Even though the averaged connectivity patterns for patients and controls were indistinguishable at the early subacute stage, the heterogeneity in lesion locations for different patients might result in idiosyncratic shifts in connectivity patterns that in the whole group would be reflected as higher variability in patterns. To measure this within-group variability, we calculated the average Euclidean distance of each patient’s pattern to the patient group mean pattern and did likewise for controls. The average within-patient distance was 2.955, whereas the average within-control distance was 2.813, resulting in a difference of 0.142 (Δvariability). We compared this value to a null distribution of Δvariability generated with permutation testing. We found that resting-state connectivity patterns of patients showed a higher idiosyncratic, nonsystematic variability compared with controls, see Fig. 4. Note that the distribution was not symmetric around zero, as the *n* for controls was smaller than for patients.

The difference in variability for intrahemispheric ipsilesional and interhemispheric connections was also higher for patients (intrahemispheric ipsilesional: Δvariability = 0.091, *P* = 0.0048; interhemispheric: Δvariability = 0.1, *P* = 0.0004, Fig. 4). In contrast, for intrahemispheric contralesional connections, we found lower variability in patients than the controls: Δvariability = −0.01, *P* < 0.0001.

### There Were No Changes in Patients’ Connectivity Patterns over Time

Even though there were no systematic differences between connectivity patterns of patients and controls at the early subacute stage, we might expect to find changes in patient connectivity patterns over time as they recover from impairment.

**Figure 5.**
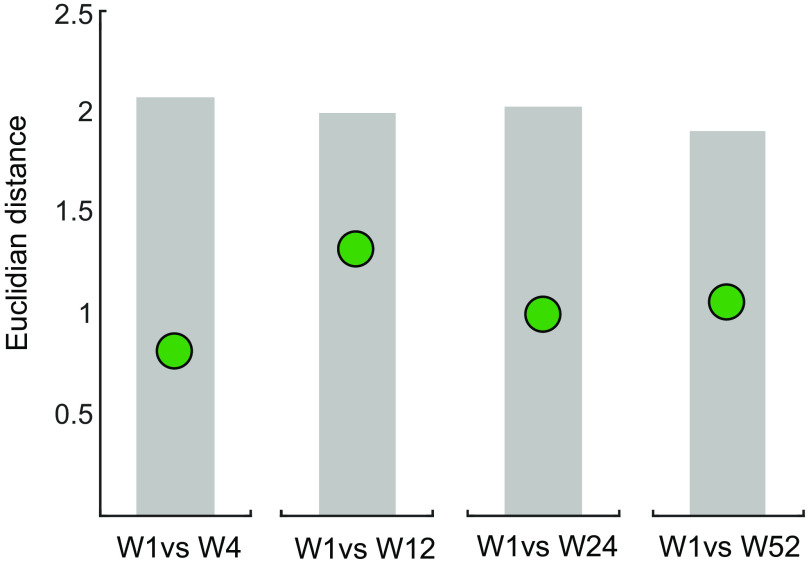
No significant change from patients’ *W1* connectivity pattern compared with time-points at the chronic stage. The green circles show the Euclidean distance between the average connectivity pattern of patients at *W1* and the average patterns for patients of all consecutive weeks (*W4*, *W12*, *W24*, and *W52*). The lower 95th percentile of the distribution under the null hypothesis of no differences between the two weeks is indicated in the gray shaded area.

We therefore quantified Euclidean distances between the average connectivity patterns at *W1* as reference versus all other weeks (Δweek). Surprisingly, patients showed no increase in Euclidean distances between *W1* and consecutive weeks (Fig. 5 and Table 2).

**Table 2.** Euclidean distances between the connectivity pattern at W1 compared with all subsequent time-points in patients for only interhemispheric, intrahemispheric ipsilesional, or contralesional subsets

Patients	*W1*_*W4*	*W1*_*W12*	*W1*_*W24*	*W1*_*W52*
All connections	Δweek: 0.814 (*P* = 0.537, Δ* > 0.68, *d* = 0.233)	Δweek: 1.322 (*P* = 0.138, Δ* > 1.3, *d* = 0.481)	Δweek: 0.994 (*P* = 0.334, Δ* > 0.92, *d* = 0.310)	Δweek: 1.063, (*P* = 0.32, Δ* > 0.98, *d* = 0.389)
Interhemispheric	Δweek: 0.636 (*P* = 0.337, Δ* > 0.7, *d* = 0.318)	Δweek: 1.018 (*P* = 0.298, Δ* > 0.74, *d* = 0.384)	Δweek: 0.665 (*P* = 0.624, Δ* > 0.45, *d* = 0.193)	Δweek: 0.77 (*P* = 0.557, Δ* > 0.54, *d* = 0.268)
Intrahemispheric ipsilesional	Δweek: 0.353 (*P* = 0.07, Δ* > 0.8, *d* = 0.595)	Δweek: 0.413 (*P* = 0.141, Δ* > 0.7, *d* = 0.561)	Δweek: 0.457 (*P* = 0.504, Δ* > 0.42, *d* = 0.298)	Δweek: 0.558 (*P* = 0.051, Δ* > 0.89, *d* = 0.711)
Intrahemispheric contralesional	Δweek: 0.367 (*P* = 0.063, Δ* > 0.73, *d* = 0.552)	Δweek: 0.735 (*P* = 0.114, Δ* > 0.65, *d* = 0.539)	Δweek: 0.579 (*P* = 0.975, Δ* > 0.01, *d* = 0.007)	Δweek: 0.474 (*P* = 0.837, Δ* > 0.23, *d* = 0.189)

As it could be expected from these results, patients showed reliably high correlations of their connectivity patterns with controls at the subacute or chronic stage (*W4*: *R* = 0.74, *P* < 0.0001; *W12*: *R* = 0.76, *P* < 0.0001; *W24*: *R* = 0.87, *P* < 0.0001; *W52*: *R* = 0.80, *P* < 0.0001) and no significant difference to control patterns (Table 3). The analyses for intrahemispheric or interhemispheric connections alone found the same result (Tables 3 and 4).

**Table 3.** Difference between the connectivity pattern of patients compared with controls at week 4, week 12, week 24, and week 52 for only interhemispheric, intrahemispheric ipsilesional, or contralesional subsets

Patients versus Controls	*W4*	*W12*	*W24*	*W52*
All connections	Δpattern: 1.203 (*P* = 0.321, Δ* > 1.14, *d* = 0.384)	Δpattern: 1.795 (*P* = 0.056, Δ* > 1.8, *d* = 0.678)	Δpattern: 0.885 (*P* = 0.577, Δ* > 0.72, *d* = 0.256)	Δpattern: 1.653 (*P* = 0.068, Δ* > 1.65, *d* = 0.669)
Interhemispheric	Δpattern: 1.102 (*P* = 0.2, Δ* > 1.08, *d* = 0.486)	Δpattern: 1.671 (*P* = 0.126, Δ* > 1.11, *d* = 0.536)	Δpattern: 0.663 (*P* = 0.822, Δ* > 0.37, *d* = 0.169)	Δpattern: 1.354 (*P* = 0.137, Δ* > 0.99, *d* = 0.524)
Intrahemispheric ipsilesional	Δpattern: 0.412 (*P* = 0.138, Δ* > 0.86, *d* = 0.635)	Δpattern: 0.44 (*P* = 0.43, Δ* > 0.52, *d* = 0.399)	Δpattern: 0.404 (*P* = 0.897, Δ* > 0.18, *d* = 0.13)	Δpattern: 0.802 (*P* = 0.066, Δ* > 0.88, *d* = 0.738)
Intrahemispheric contralesional	Δpattern: 0.253 (*P* = 0.061, Δ* > 1.04, *d* = 0.775)	Δpattern: 0.486 (*P* = 0.311, Δ* > 0.61, *d* = 0.478)	Δpattern: 0.415 (*P* = 0.794,Δ* > 0.31, *d* = 0.214)	Δpattern: 0.505 (*P* = 0.693,Δ* > 0.36, *d* = 0.307)

**Table 4.** Difference in connectivity pattern variability in patients over time for all connections, interhemispheric, intrahemispheric ipsilesional, or contralesional subsets

Patients	*W1*_*W4*	*W1*_*W12*	*W1*_*W24*	*W1*_*W52*
All connections	*F*(3,36) = 0.09, *P* = 0.9678			
	Δweek_variability: 2.47 ± 1.6	Δweek_variability: 2.61 ± 1.022	Δweek_variability: 2.42 ± 0.96	Δweek_variability: 2.64 ± 0.78
Interhemispheric	*F*(3,36) = 0.15, *P* = 0.928			
	Δweek_variability: 1.9 ± 1.4	Δweek_variability: 2.06 ± 0.782	Δweek_variability: 1.78 ± 0.697	Δweek_variability: 1.932 ± 0.61
Intrahemispheric ipsilesional	*F*(3,36) = 0.25, *P* = 0.859			
	Δweek_variability: 1.1 ± 0.524	Δweek_variability: 1.07 ± 0.529	Δweek_variability: 1.21 ± 0.485	Δweek_variability: 1.23 ± 0.461
Intrahemispheric contralesional	*F*(3,36) = 0.32, *p* = 0.814			
	Δweek_variability: 1.08 ± 0.688	Δweek_variability: 1.13 ± 0.547	Δweek_variability: 1.08 ± 0.518	Δweek_variability: 1.28 ± 0.352

By examining Euclidean distances between the individual connectivity patterns to the average connectivity pattern, we found greater nonsystematic variability in patients than in controls at *W1* and all subsequent time-points (*W4* Δvariability = −0.156, *P* < 0.0001; *W12* Δvariability = −0.109, *P* < 0.0001; *W24* Δvariability = 0.1917, *P* = 0.0002; *W52* Δvariability = 0.007, *P* = 0.016). However, this variability of patients did not change across time-points (Table 4). That is to say, there was no evidence for recovery in the form of converge on to the mean pattern over time.

In summary, we found no evidence for a mean difference of connectivity patterns between patients within 1 year. More importantly, patients did not show any significant longitudinal change in connectivity patterns either systematically or regarding their group variability.

### Comparison between Alternative Metrics for M1-M1 Connectivity Showed No Changes over Time

In our main analysis (see previous two sections), we looked at the entire connectivity pattern between five sensorimotor areas within and across hemispheres and found no changes for patients either longitudinally or when compared with controls. In contrast, some previous studies have focused on individual ROI-to-ROI connections and found changes after stroke (46). Specifically, changes in interhemispheric connectivity between the two motor cortices have been frequently reported (14–16, 47).

**Figure 6.**
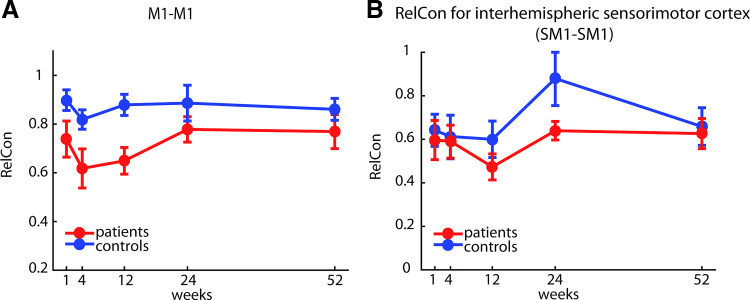
*A*: M1-M1 connectivity in our data set. In patients, interhemispheric connectivity between the two motor cortices was systematically lower than compared with controls at all time-points. However, no changes of M1-M1 connectivity over time were found. *B*: relative connectivity (RelCon) of SM1-SM1 in controls and patients. Although there was a significant difference in SM1-SM1 connectivity between the two groups, with lower RelCon for patients, there was no significant change over time.

To compare our results to these previous ones, we therefore investigated changes in interhemispheric M1-M1 connectivity weights over time and between patients and controls in our data set. A mixed-effects model showed a significant difference between patients and controls, averaged over time points, with patients having a slightly lower average correlation between motor cortices [Fig. 6*A*; mixed model, group effect*:* χ^2^(1) = 5.759, *P* = 0.016]. Congruent with our other results, however, we found no longitudinal changes in interhemispheric connectivity either when conducting a mixed model for an effect of week for the patients [χ^2^(4) = 5.836, *P* = 0.212] or for the controls [χ^2^(4) = 4.723, *P* = 0.317].

Our results also appear to contradict those in another published study, which used an alternative metric of connectivity to assess changes in functional connectivity after stroke. Golestani et al. (16) used a relative connectivity (RelCon, see methods) measure between the two sensory-motor cortices and reported lower relative interhemispheric sensorimotor (SM1 RelCon) connectivity in stroke patients with a motor deficit compared with controls and stroke patients without such a deficit.

Similarly, our patients had a lower RelCon for SM1-SM1 compared with controls at all time-points. Using a mixed model, we found a significant difference between the groups [χ^2^(1) = 5.2457, *P* = 0.022]. Again, consistent with our results reported in the previous section, we did not find a change over time for RelCon SM1-SM1 in either controls [χ^2^(4) = 2.8087, *P* = 0.5903] or in patients [χ^2^(4) = 8.2243, *P* = 0.0837; Fig. 6*B*].

## DISCUSSION

Here, we report that there were no longitudinal changes in either the mean or variability of resting-state functional connectivity (rsFC) between cortical motor areas despite substantial motor recovery over the same period in a cohort of patients with subcortical stroke. In addition, at no stage of recovery were rsFC patterns different from healthy controls.

A large number of animal studies, in rodents and nonhuman primates, have described numerous structural/physiological changes in cortical areas around and beyond the infarct core. These changes have collectively been called reorganization, but in only a small subset of cases they have been correlated with motor recovery, which suggests that most are likely just reactive (48). We reasoned that if spontaneous biological recovery is similar for cortical and subcortical strokes (49), then motor-recovery-related cortical reorganization, if not just reactive, should still occur in patients with isolated subcortical lesions. Indeed, we know that corticospinal integrity assessed with transcranial magnetic stimulation is a good predictor of recovery in patients with subcortical stroke (50, 51), i.e., cortical output is required for recovery from subcortical stroke just like it is for cortical stroke. In addition, changes in cortical maps are seen not only with cortical lesions but also with spinal and peripheral injuries (4, 52, 53).

As invasive investigations such as those performed in animal models are not available in humans, noninvasive imaging methods such as resting-state fMRI have been used to indirectly measure cortical reorganization after stroke. Here, however, we found no evidence for systematic rsFC changes between cortical motor regions. Our results are in line with several other studies that failed to find a connection between functional connectivity changes and motor outcome after stroke. Although connectivity changes at the early subacute stage could explain variance in impairment and predict outcome for language, attention, and memory deficits, motor function was best associated with lesion location and not connectivity changes (54, 55). In light of these results, previously reported cortical connectivity changes could be reactive rather than reparative, e.g., confounded by the presence of a cortical lesion.

Although our results are congruent with similar observations in a smaller, more heterogeneous cohort (17), they are seemingly contradicted by a recently published paper that reported results for resting-state changes in a similarly sized cohort of patients with subcortical stroke. In this study, Lee et al. (56) obtained six connectivity measures between 40 supratentorial and infratentorial ROIs in 21 patients with stroke assessed at two time-points after stroke (2 wk and 3 mo) and found differences in two of the measures. Specifically, they found lower overall strength in interhemispheric connectivity and higher network distance compared with controls at 2 wk, but neither changed at 3 mo. Their results taken at face value, overlooking their multiple comparisons problem and the fact that they had more variables (6 measures, 40 ROIs) than subjects, showed no connectivity measure changing over time as the patients improved, which is, in fact, entirely consistent with our results.

The question must now be asked why it was ever conjectured that changes in connections between cortical regions would enhance recovery from hemiparesis, which is caused by the interruption of descending pathways out of a particular region(s). One could rephrase this to ask Why would there be a “horizontal” solution to a “vertical” problem? This question is related to the increasing awareness of the questionable relevance of cortical map changes to recovery (4), changes that have hitherto been taken as electrophysiological evidence for reorganization (1, 57, 58). Specifically, map changes have been shown to dissociate from performance; they may be a marker for required recovery-related changes but are not causal for execution of the recovered performance (59, 60). This is of direct relevance to our current findings because a potential objection might be that if initial connectivity is not different between patients and controls, then how could subsequent changes in connectivity relate to recovery? This objection, however, conflates performance and recovery. For example, corticospinal tract integrity is correlated with current level of impairment; this same measure cannot also independently predict subsequent recovery, which presumably requires changes elsewhere that allow facilitation of residual descending pathways. Another example of an apparent dissociation between areas related to change and those related to performance is in the case of normal motor learning. It has long been known that changes occur in the basal ganglia and cerebellum with motor learning, but these structures are not responsible for the motor command that generates either the baseline or learned limb movement. Thus, map changes, corticospinal tract integrity, and normal motor learning make it clear that neural substrates for change can dissociate from those for performance. The critical question is What are the neural substrates for change when it comes to motor recovery after a lesion to the corticospinal tract?

Overall, it is increasingly apparent both from recent and previous work in nonhuman primates and rodents that motor recovery poststroke relates to changes in the strengths of descending projections to the brainstem and spinal cord from individual motor cortical areas rather than to changes in the connections between them (61–64). That said, it could be postulated that cortico-cortical drive, for example, of premotor cortex onto primary motor cortex (M1) could facilitate remaining corticospinal tract descending projections out of M1, as studies have shown such cortico-cortical facilitation in healthy nonhuman primates (27, 65). However, consistent with what we found here, there is little evidence for this as a motor recovery mechanism after stroke in any animal.

Whenever results are negative, concerns will be raised about the power and sensitivity of the study and the biological validity of the method in general.

There have been over 500 rs-fMRI studies of brain connectivity (66). Recent reports have described the close relationship between resting-state networks and structural connectivity assessed with other methods, e.g., diffusion tensor imaging (10, 67). Most notably for our purposes, the sensitivity of rsFC to changes in experience-dependent neural plasticity appears to be quite high, as even short periods of training yield statistically significant changes in functional connectivity in small *n* studies in healthy and neurologically impaired subjects (68–70). Although these previously demonstrated effect sizes might be overestimates, one would suspect that even moderate levels of sensitivity should be able to detect underlying differences in neural plasticity, given the considerable changes in motor function observed during recovery. If we look, for example, at other neurophysiological signals (e.g., EPSPs, MEPs, fMRI activation), changes in these can be quite large for subtle behavioral effects. What we do not tend to see, is the converse, subtle physiological changes in the motor system that lead to large changes in behavior.

Methodological problems with, e.g., regard to reproducibility of imaging analysis in general and rs-fMRI, in particular, have long been a topic of discussion (71, 72). So far, there is no consensus about the optimal way to analyze rs-fMRI data, which poses a fundamental challenge regarding the generalizability and comparability of results. Another methodological issue arises from data quality itself. Current studies suggest that data reliability could be improved with longer scan durations, e.g., over and above >10 min (73). In the face of a low signal-to-noise ratio, missing consensus in analysis steps and statistical methods (promoting the risk of conscious and unconscious p-hacking; 74), and frequent absence of an a priori hypothesis (which can lead to so-called HARKing; 75), the imaging literature is especially vulnerable to false-positive or false-negative results (76).

We addressed these problems by providing measures of data reliability, comparing two different preprocessing procedures, and by reanalyzing our data set with regard to individual M1-M1 changes using a previously reported metric for resting-state imaging analysis (16). To increase the transparency and reproducibility of our findings, the complete data set is available upon request and the complete connectivity maps as well as the custom-written MATLAB and R scripts are made publicly available to invite further analysis. To avoid problems, HARKing, we deliberately restricted our analysis to only include ROIs that are known to be relevant for motor function, have been hypothesized to play a role in motor recovery, and are directly or indirectly connected to corticospinal projections.

We conclude that to the extent that reorganization mediates motor recovery after stroke, it is likely attributable to upregulation of residual descending pathways and associated changes in brain stem nuclei, rather than changes in cortico-cortical connectivity. Novel noninvasive imaging and physiological approaches will be needed to capture this kind of functional reorganization.

## DATA AVAILABILITY

Custom-written MATLAB and R scripts can be found at https://github.com/MeretBran/smarts_restingstate.

## GRANTS

This work was primarily funded by the James S. McDonnell Foundation (PI: J. W. Krakauer) and the Puhringer Foundation (PI: A. Luft) and also by Wellcome Trust Grant 094874/Z/10/Z (J. Diedrichsen).

## DISCLOSURES

No conflicts of interest, financial or otherwise, are declared by the authors.

## AUTHOR CONTRIBUTIONS

P.C. and J.W.K. and conceived and designed research; M.B., J.X., and M.W. performed experiments; M.B., N.E., and C.H-C. analyzed data; M.B., J.D., and J.W.K. interpreted results of experiments; M.B. prepared figures; M.B. drafted manuscript; M.B., N.E., J.X., M.W., M.D.H., J.C.C., T.K., P.C., J.D., A.L., and J.W.K. edited and revised manuscript; M.B., N.E., J.X., M.W., M.D.H., J.C.C., T.K., P.C., C.H-C., J.D., A.L., and J.W.K. approved final version of manuscript.

## ENDNOTE

At the request of the authors, readers are herein alerted to the fact that additional materials related to this manuscript may be found at https://github.com/MeretBran/smarts_restingstate. These materials are not a part of this manuscript and have not undergone peer review by the American Physiological Society (APS). APS and the journal editors take no responsibility for these materials, for the website address, or for any links to or from it.
